# Use of Field Based Loop Mediated Isothermal Amplification (LAMP) Technology for a Prevalence Survey and Proof of Freedom Survey for African Swine Fever in Timor-Leste in 2019

**DOI:** 10.3389/fvets.2021.672048

**Published:** 2021-06-21

**Authors:** Dianne E. Phillips, Peter T. Mee, Stacey E. Lynch, Felisiano da Conceição, Joanita Bendita da Costa Jong, Grant T. Rawlin

**Affiliations:** ^1^Agriculture Victoria, Biosecurity and Agriculture Services, Bairnsdale, VIC, Australia; ^2^Agriculture Victoria Research, AgriBio Centre for AgriBioscience, Bundoora, VIC, Australia; ^3^Ministry of Agriculture and Fisheries, Government of Timor-Leste, Dili, Timor-Leste

**Keywords:** pigs, African swine fever, LAMP, prevalence, Timor-Leste

## Abstract

African Swine Fever (ASF) has been spreading in numerous southeast Asian countries since a major incursion in mainland China in 2018. Timor-Leste confirmed an outbreak of ASF in September 2019 which resulted in high mortalities in affected pigs. Pigs in Timor-Leste are the second most common type of livestock kept by villagers and represent a traditionally important source of income and prestige for householders. In order to understand the extent of ASF infected villages in Timor-Leste a prevalence survey was designed and conducted in November-December 2019. Timor-Leste has limited laboratory facilities and access to qPCR diagnostic tests. Therefore, a loop mediated isothermal amplification (LAMP) assay was used to detect ASF positive blood samples collected during the prevalence survey. The LAMP assay was proven to be a robust, highly specific and sensitive laboratory test for ASF suitable for use in the field and where there are limited laboratory facilities. The results of the prevalence survey allowed the extent of the ASF incursion to be delineated and the introduction of a disease response strategy to limit the spread of ASF and assist in the recovery of the pig population in Timor-Leste.

## Introduction

African swine fever in pigs, caused by African swine fever virus (ASFV), has been spreading through countries in southeast Asia since a major incursion in mainland China in August 2018 (1). During the reporting period from late June-early July 2020 11 Asian countries reported new or ongoing ASF outbreaks to the World Organization for Animal Health, formerly the Office International des Epizooties (OIE), with losses of 16,894 pigs (2). ASFV is the only member of the genus *Asfivirus* family *Asfarviridae* and current outbreaks have resulted in high mortalities of 80–100% in affected pig herds (3). Transport of infected pigs is a common route of disease transmission. However, the virus is also able to survive for extended periods in infected carcasses, uncooked meat products and environments or equipment contaminated by infective material, resulting in disease transmission and new incursions across much greater distances, including across international boundaries (1, 3).

Pigs in Timor-Leste are the second most common type of livestock kept by villagers in Timor-Leste. Numbers per holding are typically small (usually 1–4) and most commonly <10 animals (4, 5). They are an important source of income and prestige for villagers and are used and traded in traditional celebrations and other gatherings and exchanges (4). The species of pigs kept is typically the Timorese Warty Pig (*Sus celebensis timoriensis*) rather than the European species *Sus scrofula*. The impact of the mortalities associated with an uncontrolled ASF incursion is high in traditional villages, as there is frequently no ready source of alternative incomes and/or industry support (4, 6, 7).

Timor-Leste reported its first incursion of ASF to the OIE in late September 2019, after samples collected from sick and dead pigs around Dili were transported to the Australian Animal Health Laboratory in Geelong, Australia, where they tested positive to ASFV using real time PCR (8). The initial outbreak information consisted of reports of 100 small holder outbreaks resulting in 405 dead pigs around Dili, and unconfirmed reports of pig deaths in two other northern municipalities, Baucau and Liquicia. A survey of animal health staff in all Timor-Leste municipalities undertaken by Food and Agriculture Organization of the United Nations (FAO) during Oct and November also reported 21,155 dead pigs and 7,335 sick pigs (unpublished data).

OIE notes in its recommended measures for an ASF outbreak that control of ASF can be difficult and must be adapted to the specific epidemiological situation (3). The classic stamping out and eradication response used in countries such as Australia (9) may not be practical or implementable due to epidemiological and societal factors, logistical, technical and financial limitations. In particular, diagnostic capacity must be considered, given that several other diseases of pigs are endemic in Timor-Leste, including Classical Swine Fever, that cannot be differentiated from ASFV on clinical signs (1, 3). In this outbreak, it was unclear what proportion of the FAO survey results were attributable to ASFV. Timor-Leste has limited laboratory facilities and staff and at the time of the ASF incursion, did not include any diagnostic tests for ASF or CSF. In addition, once ASF was confirmed in the country, quarantine restrictions made it very difficult for Timor-Leste to send any more diagnostic samples to an animal health laboratory in another country such as Australia.

**Figure 1:**
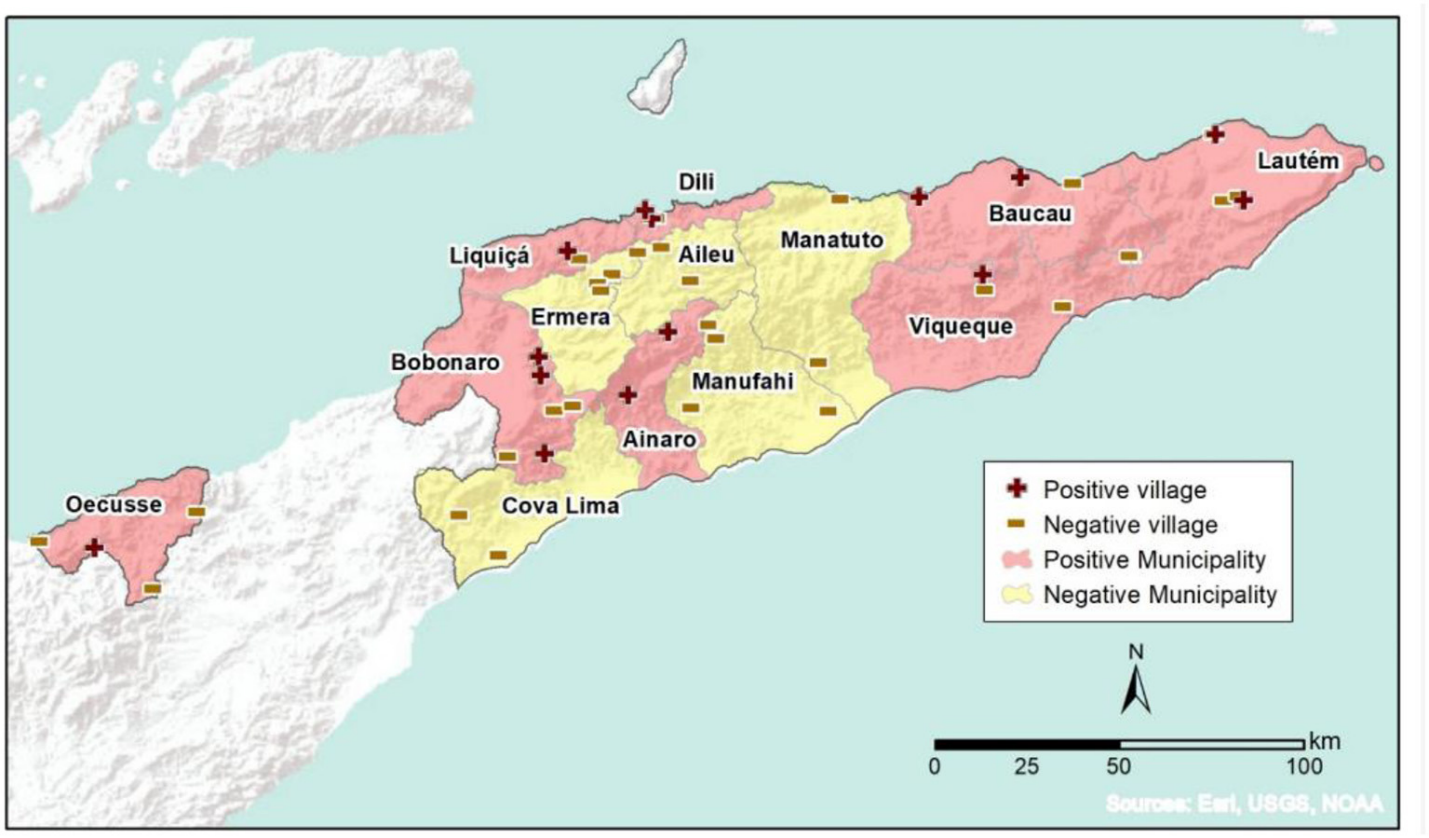
Map of mainland prevalence survey results.

In order to decide on an appropriate response to the ASF outbreak, a project team from Australia worked with the Timor-Leste animal health services to deliver a number of outputs. A disease prevalence survey was designed to delineate the extent of the incursion on the mainland. Secondly, where there were areas that may have been free of disease a proof of freedom study was undertaken. Given preliminary disease reports were primarily from coastal areas on the mainland, the team hypothesized that two outlying areas may still be free of ASF: the municipality of Oecusse [embedded within West Timor, which at that point had not recorded any cases of ASF (10)] and Atauro Island some 25 km north of the mainland coastline, near Dili (see Figure 1). Atauro Island has fewer opportunities for disease spread, however it is serviced by a ferry and charter boats from Dili and a ferry from Oecusse. Pigs are transported on ferries in both directions. Since the ASF outbreak there had been limited reports of sudden deaths in pigs on the island and, if proven to be free of ASF, the island could serve as an important source of ASF free pigs for future breeding.

Finally, the project team sought to verify whether the use of LAMP technology would prove a suitable method of laboratory diagnosis in the ASF outbreak within Timor-Leste. After training was provided to the Timor-Leste animal health laboratory staff, samples from ASFV infected pigs to were tested using an ASFV specific, loop mediated isothermal amplification (LAMP) assay for the rapid diagnosis of ASF in the field or in basic laboratory facilities. The incorporation of a fit for purpose laboratory test, especially where multiple disease etiologies are present and indistinguishable at a clinical or gross pathology level, is also an essential step planning an emergency animal disease response (3). In countries where animal health laboratories have limited resources and skilled personnel, the use of a LAMP assay presents a viable alternative, or addition to existing recognized laboratory tests (11, 12).

## Materials and Methods

### Mainland Prevalence Survey

Pigs are largely farmed in at a subsistence level in most of Timor-Leste, including rural villages and peri-urban households. Most families in a village own a few pigs and the pigs are held in small pens or tethered near village houses. Biosecurity practices are limited with regards to use of personal protective equipment for pig owners and cleaning and disinfection of equipment used to house and feed pigs. Feed sources for pigs commonly include leftover food from households and/or pigs are allowed to free range in the village where they can access food scraps and/or rubbish (4, 6). Therefore, it was considered probable that if a pig in a village became infected with ASFV it was likely that multiple pigs within the village would become infected in a short period of time and that a village with ASFV infected pigs represented a suitable unit of interest for the prevalence survey design.

Timor-Leste conducts a population and livestock census every 5 years and the results of the census from 2015 were used in planning the prevalence survey (5). Using Epitools Sample size to estimate a simple proportion (apparent prevalence) online calculator (13) and the following assumptions:

1. Assumed prevalence of ASF 0.1.
2. Desired type I error = 0.05.
3. Desired type II error = 0.05.
4. Source population 422 (all villages in Timor-Leste except Atauro Island and Oecusse).

The number of villages to be sampled was calculated to be 35 (modified hypergeometric exact calculation). Using the census village data, the sample number was proportionally distributed according to the number of villages in each municipality. Random villages were selected for sampling using a random number generator across each municipal village sampling frame. If the selected village was not logistically possible to sample, the next village within a 10 km radius of the original village was selected (Table 1).

**Table 1:** Timor-Leste municipalities and randomly selected villages for mainland ASFV prevalence survey.

**Municipality**	**Number of villages in municipality**	**Proportional adjusted number of villages to sample**	**Random number selected village for sampling**
Aileu	32	3	Fatisi Liquidoe Madabeno
Ainaro	21	2	Ainaro Maubisse
Baucau	60	5	Lasula Tequinomata Laisorolai Lour Betalale
Bobonaro	50	4	Bobonaro Caribau Guda Lebos
Covalima	30	2	Lactos Maudemu
Dili	26	2	Comoro Vila Verde
Ermera	52	4	Laclo Tiarlelo Talimoro Letefoho
Lautem	34	3	Euquisi Parlamento Raça
Liquicia	23	2	Leorema Metagou
Manatuto	29	2	Manehat Ma'Abat
Manufahi	29	2	Beremana Caimau
Viqueque	36	3	Ossu Afaloicai Bahatata
Total	422	35	35

Epitools online calculator (14) was used to calculate the true disease prevalence at a village level. Assumptions for the calculation were the LAMP test sensitivity (Se) 0.98 and test specificity (Sp) 0.999 (15) and a confidence level of 0.95. The calculation tool recommends the use of Blaker's interval for confidence levels for general use.

### Proof of Freedom Sample Design for Atauro Island

The following assumptions were used to calculate a sample size for proof of freedom from ASF on Atauro Island:

1. Population size = 5,000 (5).
2. Test Sensitivity = 0.98 (based on preliminary unpublished LAMP test data).
3. Test Specificity = 0.999 (based on preliminary unpublished LAMP test data).
4. Design prevalence to detect disease at *P* = 0.1.
5. Desired type I error = 0.05.
6. Desired type II error = 0.05.

Using Epitools 1-stage freedom analysis (16) and a modified hypergeometric calculation, the number of random samples required is 29. (If a random sample of 29 units is taken from a population of 5,000 and 0 or fewer reactors are found, the probability that the population is diseased at a prevalence of 0.1, *p* = 0.05).

Epitools online calculator for a 1-Stage Freedom Analysis was used to analyze the test results (16).

### Proof of Freedom Survey for Oecusse Municipality

In the interval between planning and collection of samples (December 2019), Indonesia reported its first cases of ASF (17) and instead of conducting a proof of freedom survey for the municipality of Oecusse, it was considered to be likely to at high risk, or already have ASF infection as it is surrounded by land borders with Indonesia. This municipality was subsequently included in the mainland prevalence survey instead.

### Village Sampling Visits

Within each village, three households with pigs were visited. Those with sick or recently dead pigs were targeted and up to 5 pigs per household were examined and sampled. If more than 5 pigs were present in a household, pigs were selected if there was a history of ill health, or clinical signs of poor condition, or a selection of pigs to cover ages and different pens was used. Pigs were restrained manually or using a pig snare and a cranial vena cava blood sample was collected in two plain blood tubes. Where possible, the pigs' rectal temperature was recorded and if the owner reported the pig was sick, or it had an elevated temperature, an oral and rectal swab was collected as well. For each household where pigs were sampled, the number of pigs at risk, sick pigs and dead pigs within the last month was recorded.

Analysis of the association between the presence of pyrexia (Pigs with rectal temperatures of 40.0C or higher) and a positive LAMP ASF test was conducted using a 2 × 2 risk table and Epitools online calculator incorporating a 95% confidence level and a Fisher's exact test (2-tailed) (18).

The pig samples were stored in a car fridge at 4C until they could be tested using the ASFV LAMP assay, either in the field or at the animal health laboratory in Dili. The time frame from pen side collection to LAMP testing varied from a few hours to 2–3 days.

Disinfection of personnel and equipment was undertaken between each village.

### Preparation of Samples and the ASFV LAMP Assay

Serum was separated from blood by centrifuging for 20 min at 3,000 g. Serum was removed from the tubes and tested straight away or stored at −20°C until testing. Samples were tested for ASFV using an ASFV LAMP assay (15). Briefly, serum was diluted 1 in 10 in nuclease-free water before 2 μL of each sample was heat treated at 95°C for 2 min. LAMP reactions were setup with 15 μL of Isothermal Mastermix ISO-DR004-DT (OptiGene Ltd., Horsham UK), 2.5 μL of primer mix targeting the topoisomerase II gene (19) with a final primer concentration of F3/B3 0.2 μM, FIP/BIP 1.6 μM and loop primers at 0.8 μM and reaction made up to 25 μL with nuclease-free water. Reactions were run on a Genie III (OptiGene, Horsham, UK), instrument with run conditions of 65°C for 25 min. Each run included a synthetic positive control as well as a no template control. A sample was classified positive if the time to positivity (T_p_) < 20 min and had an annealing temperature (T_a_) of 87.42°C (± 0.56°C).

## Results

### Mainland Prevalence Survey

Field teams collected 449 samples from 48 villages over a 3-month period from to late September 2019 to mid-December 2019. Of these 13 samples that were collected were not able to be tested due to issues relating to transport or storage or transcription errors in laboratory recording and were removed from the data set.

Of the remaining 436 samples, 59 samples tested positive to ASFV using LAMP and 377 samples were negative. There was ASFV detected in 16 villages within 8 municipalities. Across the remaining five municipalities ASFV was not detected in any of the 32 villages sampled. The distribution of positive and negative villages is shown in Table 2 and the map in Figure 1.

**Table 2:** Results of pig samples tested for mainland prevalence survey.

**Municipality**	**District**	**Village**	**Number of pigs tested**	**Number of pigs positive ASF**	**Village ASF status** **(N = negative, P = positive)**
Aileu	Aileu Vila	Fatisi	7	0	N
		Madabeno	7	0	N
	Lequidoe	Liquidoe	6	0	N
Ainaro	Ainaro	Ainaro Vila	7	2	P
	Maubesi	Maubesi Vila	7	1	P
Baucau	Vemasse	Caicua	25	4	P
		Vemasse Tasi	17	7	P
	Baguia	Larisula	10	0	N
	Laga	Tequinomata	10	0	N
	Quelecai	Laisorulai	6	0	N
		Laisorulai de Baixo	4	0	N
Bobonaro	Cailaco	Meligo	10	2	P
		Goulolo	10	7	P
		Manapa	7	6	P
		Atudara	13	2	P
	Bobonaro	Bobonaro	10	0	N
		Caribau	10	0	N
	Lolotoe	Guda	7	1	P
		Lebos	10	0	N
Covalima	Tilomar	Maudemo	10	0	N
	Fohorem	Laktos	11	0	N
Dili	Dili	Beto, Becora, Bidau, Bebonuk	35	14	P
	Vera Cruz	Vila Verde	6	0	N
	Dom Alexio	Comoro	8	2	P
Ermera	Ermera	Kokoa	7	0	N
		Talimoro	12	0	N
		Eraulu	6	0	N
		Haufu	8	0	N
Lautem	Lospalos	Fuiloro	8	0	N
		Rasa	8	3	P
		Souro	8	0	N
	Moru/Parlamento	Euquisi	6	0	N
		Parlamento	6	2	P
Liquicia	Bazartete	Metagou	13	1	P
		Leorema	10	0	N
Manatuto	Manatuto	Ma'Abat	10	0	N
	Natarbora	Manehat	10	0	N
Manufahi	Faberliu	Fatucahi	9	0	N
	Same	Babulu	6	0	N
	Turiscai	Beremana	7	0	N
		Caimauc	7	0	N
Oecusse	Pante Makasar	Nipani/Sakato	5	0	N
	Passabe	Passabe	5	0	N
	Oesilo	Bobometo	8	3	P
	Citrana	Bene-Ufe/Naktuka	10	0	N
Viqueque	Watucarbau	Bahatata	6	2	P
	Watulari	Afloicai	3	0	N
	Ossu	Ossu de sima	5	0	N
Number of negative villages					32
Number of positive villages					16
Total			436	59	48

Additional villages (above the number specified in the design survey) arose either through additional sampling undertaken by field staff in response to reports from local animal health staff of recent deaths or illness in village pigs or with the inclusion of ASF samples already collected in the initial months of the outbreak which were retested in using the LAMP machine and protocol and were included to improve the accuracy of the prevalence survey.

The 16 test-positive villages, adjusted for the assumed accuracy of the LAMP assay (Se = 0.98, Sp = 0.999) gave a village-level true prevalence of 16/48 = 34% (95% CI 22–48%).

**Figure 2:**
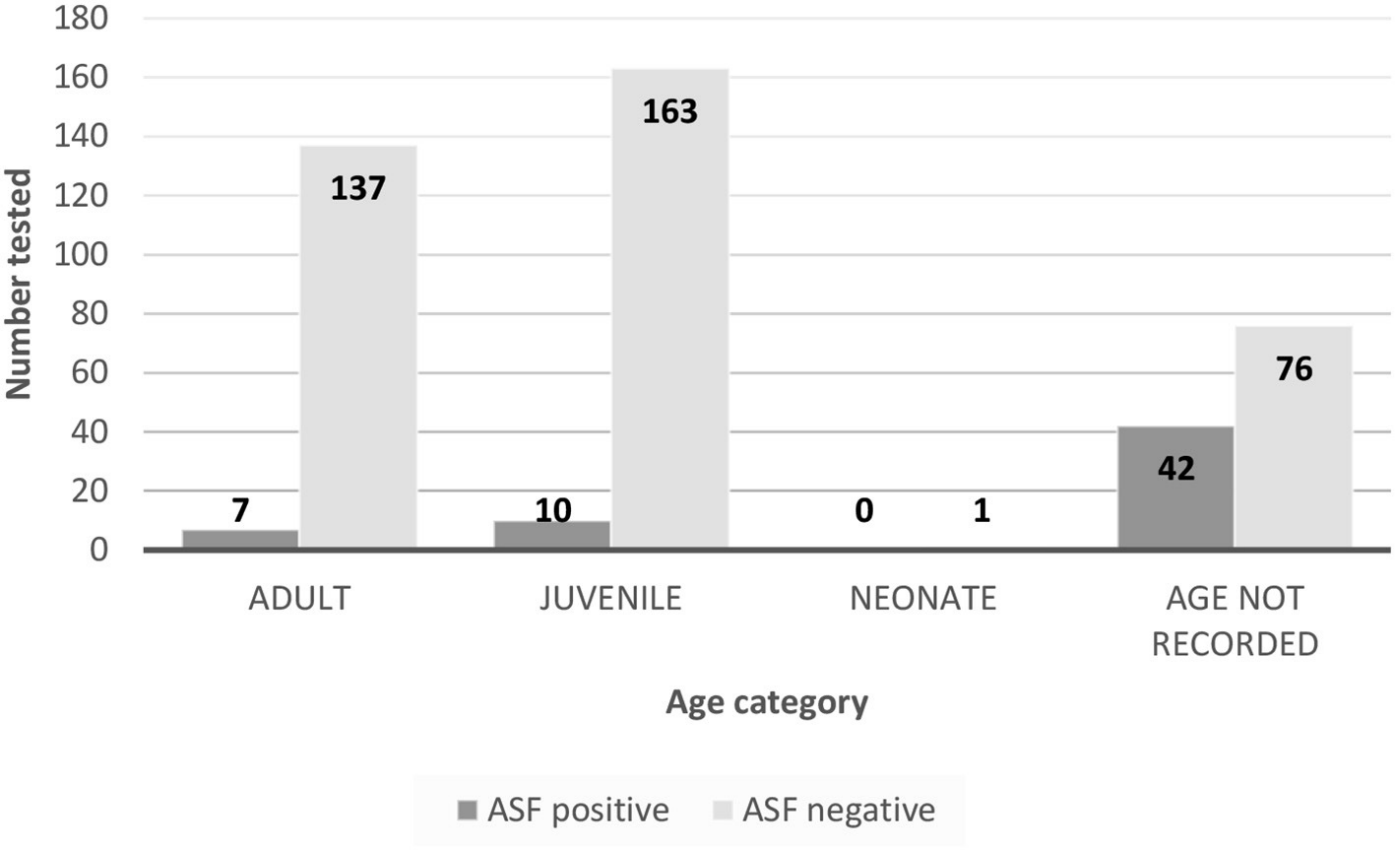
Summary of ASF test results versus age of pigs sampled for mainland prevalence and Atauro Island survey.

**Figure 3:**
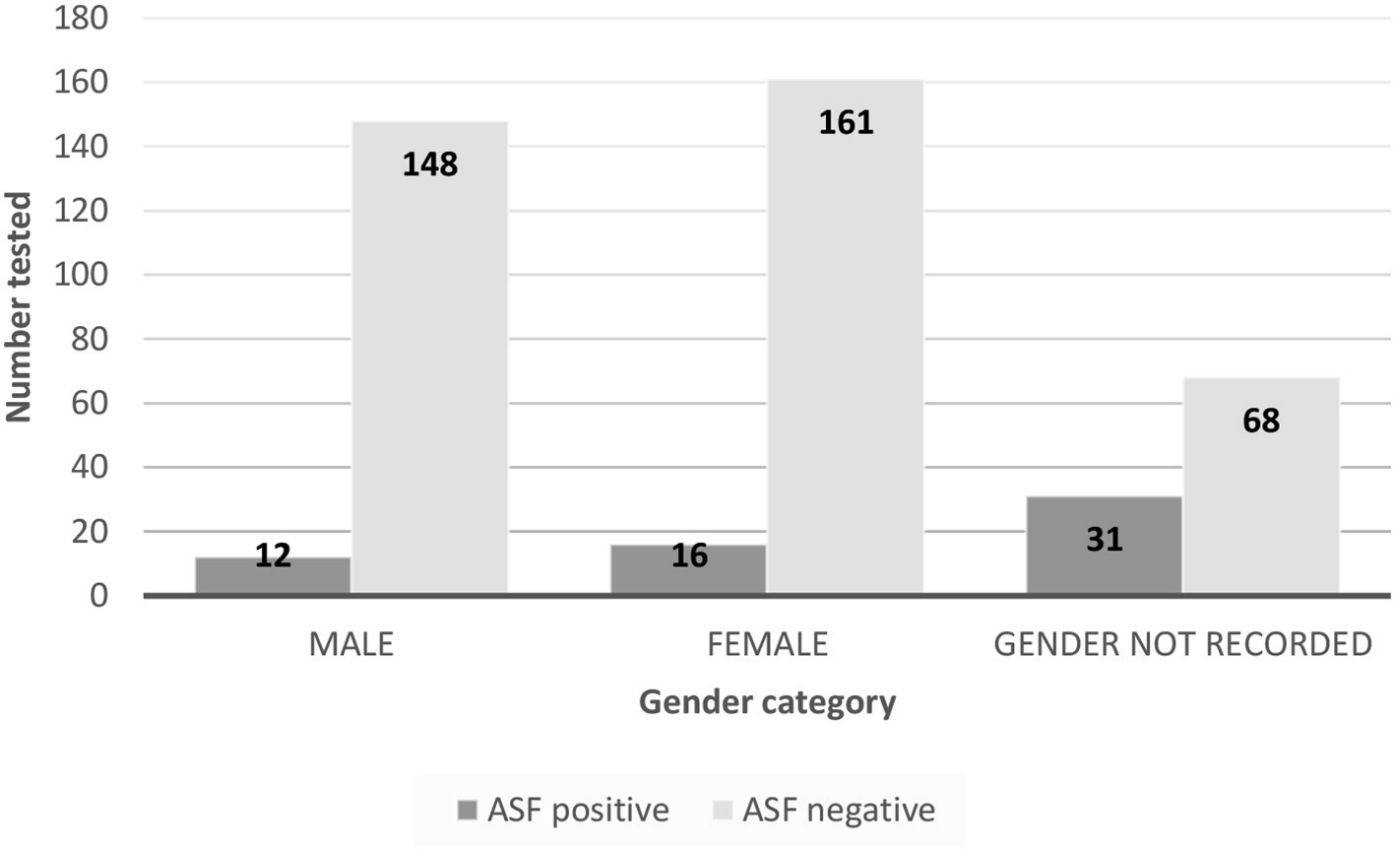
Summary of ASF test results versus gender of pigs sampled for mainland prevalence and Atauro Island survey.

Not all pig samples were accompanied by clinical records and where records were submitted, not all fields were completed. The distribution of age and gender of pigs sampled, and ASF test results are summarized in Figures 2, 3.

There were 284 records which included the rectal temperature of the pig at the point of sampling. The distribution curve of body temperature recordings was approximately normal (not shown). The incidence risk ratio for a positive LAMP test given pyrexia (Table 3) was 5.67 (95% confidence interval 1.85–17.42, *p* = 0.01).

**Table 3:** 2 × 2 risk table of pigs sampled for mainland prevalence and Atauro Island survey with or without pyrexia and ASF disease status.

**Body temperature**	**ASF positive**	**ASF negative**	**Total**
Pyrexia	4	19	23
No pyrexia	8	253	261
Total	12	272	284

### Atauro Island Proof of Freedom Survey

**Figure 4:**
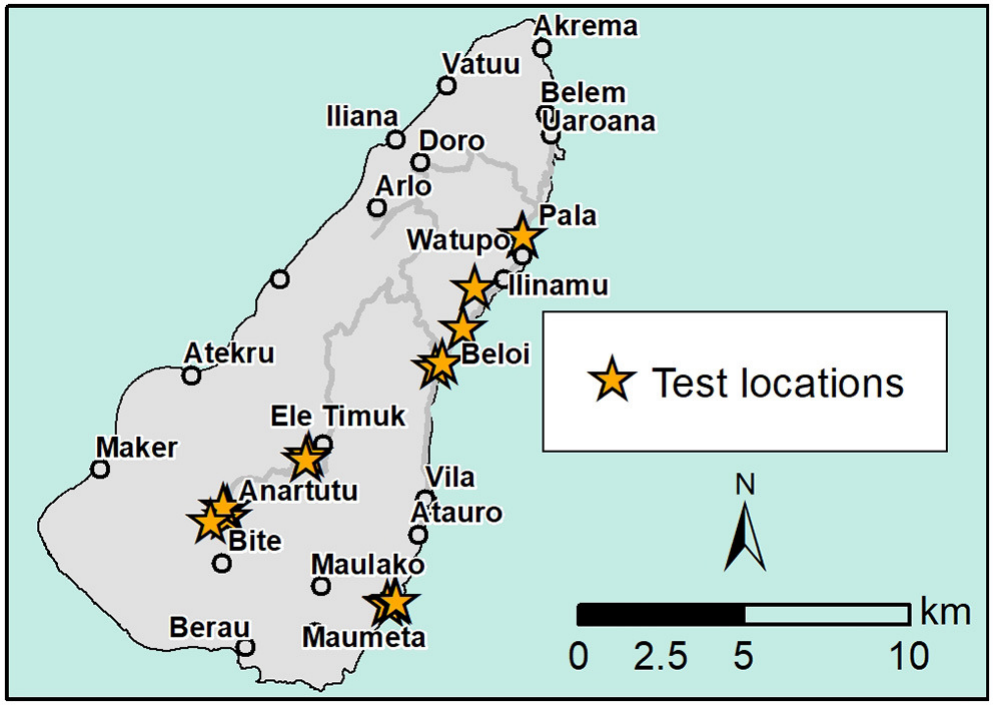
Map showing sample results and location on Atauro Island.

East Timor and Agriculture Victoria staff completed the collection of 33 random samples from pigs in several villages on the island from 7 to 8 November 2019 (Figure 4). A mixture of inland and coastal villages was sampled, and within villages, pigs to be sampled were organized by the local animal health staff with a bias toward recently sick pigs.

Testing of the samples using LAMP was completed on 9 November 2019 and all samples were negative. Figure 3 shows the map of test results.

The null hypothesis set the probability of observing no reactors in a sample of 33 individuals from a population with a disease prevalence of 10% at 0.0318. The alternative hypothesis set the probability of observing at least one reactor in a sample of 33 individuals from a disease-free population at 1. These results are adequate to reject the null hypothesis and conclude that the population is free from disease (at the expected minimum disease prevalence of 10%) at the 0.9682 confidence level.

## Discussion

The completion of the Timor-Leste mainland ASF disease prevalence survey was successful in calculating an estimate of the level of ASFV infection present at a village level and the geographic extent of the spread of disease. The infected village prevalence estimate of 34% (95% CI 22–48%) indicates that whilst approximately one third of villages in Timor-Leste have ASFV infection, the level could be as high as nearly half or as low as one quarter of all villages. A more precise estimate would be ideal but the availability of resources and logistical challenges in collecting samples from remote villages are limiting factors in Timor-Leste. The value of the survey is not however, constrained to the accuracy of the estimate, but by a reckoning of the level and distribution of disease to inform a disease response.

The Timor-Leste mainland prevalence survey described the current geographic distribution of disease. ASFV infected villages were localized mainly in the west and the east of the mainland with no detectable disease in the far south east municipality of Cova Lima or in a group of 4 municipalities to the south of the capital Dili. The prevalence survey design assumed that ASF, if present, would be detected in 10% of villages, a fairly conservative estimate given the infectious nature of the virus, the time elapsed between its detection in early September 2019 and the time of the majority of sample collection in November and December 2019 and the size of the mainland. However, it is possible that if ASFV was present in <10% of villages it would not have been detected in the numbers of villages sampled.

This prevalence survey detected discrepancies with the earlier unpublished phone survey reports of significant numbers of sick and dead pigs in most municipalities including Aileu, Manufahi, and Cova Lima. where there was no diagnostic evidence in the prevalence survey. It demonstrates the limitations of phone survey results based on reported clinical signs or without the addition of diagnostic testing to differentiate between other potential causes of sudden death and/or acute disease in pigs, including Classical Swine Fever (CSF). In this study 19 out of 23 pigs with pyrexia tested ASFV negative, suggesting other febrile causes of disease are common in Timor-Leste. CSF vaccination campaigns have been conducted by the government since 2003 but a study in 2015 estimated CSF seroprevalence at 34.4% with evidence of virus circulation and associated mortality events in village herds surveyed (20). In the CSF seroprevalence survey, pigs that has been vaccinated for CSF were more likely to have antibodies; however these pigs only accounted for a percentage of samples tested. There may also be language barriers that affect the accuracy of the information collected via phone surveys.

The first detection of disease in Timor-Leste occurred around Dili, Baucau and Liquicia (all northern coastal municipalities) (8). It is not known how ASFV was introduced but common routes of spread are noted to include movement of infected pigs or infected pork products (3). Timor-Leste has limited international transport options for live pigs via sea routes, and only one land border is with West Timor (a province of Indonesia that did had not declared an outbreak of ASF at the time of the Timor-Leste detection) so the opportunity for movement of infected live pigs into the country was limited. It is possible that there were imported ASFV infected pork products that were fed to domestic pigs or became available for local pigs to scavenge. ASFV can remain viable in fresh and frozen pork products for at least 105 days and the risk of introduction of disease via pigs accessing such pork products is well-documented (3, 21, 22). For example, between 5 November 2018 and 30 November 2019, Australian authorities intercepted over 34 tons of pork products on air travelers entering Australia (23, 24) of which a percentage (figure unpublished) tested positive for ASFV DNA. There are a significant number of foreign-aid funded capital works projects in Timor-Leste such as a new commercial port construction near Dili and road infrastructure projects that have associated risks of disease introduction via associated with foreign workers and imported machinery.

After the initial incursion of ASFV, spread of disease has occurred to the infected villages identified in this prevalence survey. Routes of spread typically involve the movement of infected pigs and contaminated pig products and/or equipment and people (1, 3, 22). In Timor-Leste cultural practices of both pig housing with minimal biosecurity and transporting pigs for traditional occasions of importance (4) could allow disease spread both at a local level within and between neighboring villages and translocation to new areas via transport of infected pigs and material via road or boat. The northern and southern municipalities of Timor-Leste are separated by a high range of mountains over 2,000 m above sea level and the few roads that cross are steep, windy and in poor condition in many places. This range may have limited the movement of pigs moving from north to south in some areas. There are police check points built on key routes that may in future be used in a disease control response to limit pig movements to restrict the spread of disease (pers.comms). Other types of transmission routes for ASFV that are of unknown significance in Timor-Leste include the role of ticks and biting insects and external parasites. External parasites including ticks and lice were observed on sampled pigs and potentially could act as vectors for ASF (3, 22). Further research is needed to elucidate the role of ticks and other biting insects in the transmission of ASFV in Timor-Leste.

ASF in pigs is commonly associated with pyrexia (3). Analysis of the association between pyrexia and the detection of ASFV showed that whilst pyrexia was a significant relative risk factor, not all ASFV positive pigs were pyrexic when sampled (*n* = 4). The logistical difficulties of blood collection from pigs in remote villages in Timor-Leste warrants the investigation of alternative sampling strategies that could be collected by less skilled local animal health staff as an alternative early detection system for ASFV and/or other diseases associated with pyrexia in pigs. However, in this study the usefulness of pyrexia as an indicator of infectious disease was limited. The use of other samples such as oral or fecal swabs are an area for future research in Timor-Leste.

The proof of freedom survey of Atauro Island gave a high level of confidence that there was unlikely to be ASF on the island at the expected minimum prevalence of 10%, at the 0.9682 confidence level. This informed an important disease control initiative based on the assumption that the pig population there was free from ASFV. The island was immediately quarantined by the Timor-Leste Ministry of Agriculture and Forestry on 8/11/2019 to prevent any further movement of pork or pigs to the island (pers.comms). Trade in pigs back to Dili remains as normal. An important consideration in Timor-Leste is that the Timor Warty Pig is only found on the islands of Timor. It is not only culturally important but is unique to the islands. Therefore, the preservation of the species is a priority. Disease free refuges of breeding stock are important for future restocking on the mainland.

Until this project was undertaken there was no diagnostic capability or capacity in Timor-Leste for ASF or other viral diseases including CSF affecting pigs. The use of LAMP technology in Timor-Leste has provided a fast, simply performed and robust test suitable for use in a basic laboratory or field situations. Test specificity and sensitivity appear very high (>99.9%) based on previous research (15) and preliminary validation work in Australia with serum samples from known ASF free animals and seeded manufactured DNA samples (unpublished data). Serum samples were used in this prevalence survey to maximize the familiarity of local field staff experience and the availability of blood tube supplies. Acknowledging that serum generally contains less viral genome than whole blood, preliminary LAMP testing was also conducted on whole blood samples in a variety of types of anticoagulants and is reported in a separate publication (15). The use of liquid reagents for the field tests undertaken with the LAMP machine created minor issues when the reagents were exposed to high ambient temperatures associated with the tropical climate in Timor-Leste. In the future, use of freeze dried reagents to increase stability in a variety of climates is expected to address this problem and future research is planned to verify that this is the case.

The completion of a prevalence survey to inform any jurisdictional response agency about disease distribution is a crucial step in planning any response to a new disease incursion (3, 9). As a result of this project, Timor-Leste has implemented a staged disease response that includes some movement controls, improving biosecurity awareness and practices amongst village pig owners, further sampling to ascertain the effectiveness of the implemented disease control measures and further research and training to enable the most effective use of available laboratory resources and in-country animal health staff.

## Data Availability Statement

The raw data supporting the conclusions of this article will be made available by the authors, without undue reservation.

## Ethics Statement

Ethical review and approval was not required for the animal study because Samples were collected as part of an ongoing ASF outbreak investigation. Written informed consent for participation was not obtained from the owners because Verbal permission was obtained from owners of livestock and is considered normal practice in Timor-Leste.

## Author Contributions

DP: design of prevalence survey, proof of freedom survey, and field work. SL and PM: diagnostic test creation, laboratory support, and field application. GR: team leader and field work. FC: field work. JB: chief veterinary officer Timor-Leste and ministerial liaison. All authors contributed to the article and approved the submitted version.

## Conflict of Interest

The authors declare that the research was conducted in the absence of any commercial or financial relationships that could be construed as a potential conflict of interest.
